# Proteomic Insights into Sulfur Metabolism in the Hydrogen-Producing Hyperthermophilic Archaeon *Thermococcus onnurineus* NA1

**DOI:** 10.3390/ijms16059167

**Published:** 2015-04-23

**Authors:** Yoon-Jung Moon, Joseph Kwon, Sung-Ho Yun, Hye Li Lim, Jonghyun Kim, Soo Jung Kim, Sung Gyun Kang, Jung-Hyun Lee, Seung Il Kim, Young-Ho Chung

**Affiliations:** 1 Division of Life Science, Korea Basic Science Institute, Daejeon 305-806, Korea; E-Mails: moonyj@kbsi.re.kr (Y.-J.M.); joseph@kbsi.re.kr (J.K.); sungho@kbsi.re.kr (S.-H.Y.); bluehyeli@naver.com (H.L.L.); kjh1052@kbsi.re.kr (J.K.); sjkim313@kbsi.re.kr (S.J.K.); ksi@kbsi.re.kr (S.I.K.); 2 Marine Biotechnology Research Division, Korea Institute of Ocean Science and Technology, Ansan 426-744, Korea; E-Mails: sgkang@kiost.ac (S.G.K.); jlee@kiost.ac (J.-H.L.); 3 Graduate School of Analytical Science and Technology, Chungnam National University, Daejeon 305-764, Korea

**Keywords:** *Thermococcus onnurineus* NA1, nano-UPLC-MS^E^, comparative proteomics, elemental sulfur, H_2_S, hydrogenases, sulfur metabolism, oxidative stress defense

## Abstract

The hyperthermophilic archaeon *Thermococcus onnurineus* NA1 has been shown to produce H_2_ when using CO, formate, or starch as a growth substrate. This strain can also utilize elemental sulfur as a terminal electron acceptor for heterotrophic growth. To gain insight into sulfur metabolism, the proteome of *T*. *onnurineus* NA1 cells grown under sulfur culture conditions was quantified and compared with those grown under H_2_-evolving substrate culture conditions. Using label-free nano-UPLC-MS^E^-based comparative proteomic analysis, approximately 38.4% of the total identified proteome (589 proteins) was found to be significantly up-regulated (≥1.5-fold) under sulfur culture conditions. Many of these proteins were functionally associated with carbon fixation, Fe–S cluster biogenesis, ATP synthesis, sulfur reduction, protein glycosylation, protein translocation, and formate oxidation. Based on the abundances of the identified proteins in this and other genomic studies, the pathways associated with reductive sulfur metabolism, H_2_-metabolism, and oxidative stress defense were proposed. The results also revealed markedly lower expression levels of enzymes involved in the sulfur assimilation pathway, as well as cysteine desulfurase, under sulfur culture condition. The present results provide the first global atlas of proteome changes triggered by sulfur, and may facilitate an understanding of how hyperthermophilic archaea adapt to sulfur-rich, extreme environments.

## 1. Introduction

Thermococcales are strictly anaerobic and heterotrophic hyperthermophiles that can utilize various complex substrates such as yeast extract, peptone, and amino acids such as carbon sources, and grow optimally at temperatures between 80 and 100 °C, depending on the species. During growth, they utilize elemental sulfur (S°) as a terminal electron acceptor for energy conservation, or as a source of sulfide for biosynthesis of sulfur-containing amino acids (Cys and Met), cofactors (e.g. Fe–S clusters, molybdopterin, thiamine), and nucleosides [1,2]. Alternatively, many species can also gain energy via fermentation of amino acids or sugars, with a coupled process producing organic acids, CO_2_, and H_2_ in the absence of S° [1,3,4]. The metabolic mechanisms, bioenergetic benefits, and the genes involved in H_2_ and sulfur metabolism have been well studied in only a few members of the Thermococcales, particularly, *Thermococcus kodakarensis* KOD1 [5,6] and the related species *Pyrococcus furiosus* [7,8,9]. Evidence from transcriptional and biochemical analysis, supported by mutational analysis data, have revealed a core set of genes involved in the S° reduction of *P. furiosus* [10,11,12]. These findings have greatly advanced our understanding of the fermentation-based S° reduction mechanism in hyperthermophilic archaea. However, sulfur metabolism in Thermococcales has mainly been studied at the genomic and transcriptomic levels, while no extensive analysis at the proteome level has yet been investigated to examine the response to sulfur. Despite intensive efforts from current genomics and transcriptomic studies, the impact of transcript regulation on actual protein levels remain poorly understood. Moreover, knowledge about the metabolic process and enzymes unique to assimilatory sulfur metabolism is still limited. Because proteins are directly responsible for cellular functions, measurements of protein abundances under different substrate culture conditions are expected to provide significant clues to the modulation of cellular functions during growth on sulfur. Proteomic profiles can potentially provide useful information about cellular functionality and mechanisms of metabolic changes in hyperthermophilic archaea. Knowing to what extent proteins are expressed and how they merge to constitute a functional unit in response to sulfur also provides relevant information to understanding how hyperthermophilic archaea responds to different environmental conditions. In addition, quantitative proteomics data can be used as a cross-reference to validate archaeal sulfur metabolism and hydrogen fermentation.

*Thermococcus onnurineus* NA1, an unusual member of the archaeal Thermococcales order isolated from a deep-sea hydrothermal vent area, is able to utilize elemental sulfur as a terminal electron acceptor for heterotrophic growth on peptides or amino acids, and reduce it to H_2_S [13]. In the absence of sulfur, it is known to efficiently produce biohydrogen (H_2_) using CO, formate, or soluble starch as the growth substrate [14]. Genome sequence analysis of *T. onnurineus* NA1 identified metabolic pathways and enzymes that are involved not only in organotrophic growth, but also in carboxydotrophic growth [15]. *T. onnurineus* NA1 is also of more general interest because it can derive enough energy from CO- and formate-oxidation coupled proton reduction to sustain growth [15,16]. Thus, due to its ability to utilize such carbon and energy sources, as well as its ability to metabolically reduce S° for heterotrophic growth, much attention has been focused on *T. onnurineus* NA1 to examine its potential for biohydrogen production. *T. onnurineus* NA1 is also assumed to have highly efficient metabolic strategies which allow it to thrive on these different growth substrates in hydrothermal vents. In addition, the CO- and formate-dependent hydrogenogenic metabolism has been extensively studied by gene disruption and transcriptional analysis. Owing to the discovery of new enzymes, several distinct hydrogenase gene clusters, and their regulatory ability, it shows great potential for use in biotechnology [17,18,19]. This metabolic versatility, together with the significant potential for biotechnology uses, would provide a good platform to study sulfur-dependent metabolic changes, as well as to examine the activities of hydrogenases, sulfur reductase, and electron transfer occurring in *T. onnurineus* NA1, at both the protein and genetic levels.

To date, complete genome sequences have been determined for a number of Thermococcales, including three representative *Pyrococcus* species [20,21,22] and four *Thermococcus* strains: *T. onnurineus* NA1 [15], *T. kodakarensis* KOD1 [23], *T. gammatolerans* EJ3 [24], and *T. sibiricus* MM739 [25] and are publicly available for *T. barophilus* MP [26] and *Thermococcus* sp. AM4 [27]. This wealth of sequence information accumulated in recent years has enabled the full-scale use of genome based techniques such as transcriptomics and proteomics for the characterization of these hyperthermophilic archaea. The growth of cells on different substrates provides the opportunity to interrogate specific physiological responses by measuring the changes in protein abundance. To date, there have only been a few published proteome studies of Thermococcales. Using 2-DE/MS-MS proteome analysis, we previously identified several proteins and metabolic pathways of *T*. *onnurineus* NA1 during heterotrophic growth, providing a framework of the mechanisms activated in response to sulfur [28]. In addition, SDS-PAGE/LC-MS/MS shotgun proteomic analysis revealed that metabolic enzymes specific for hydrogen production were prominently expressed when *T. onnurineus* NA1 cells were cultured under carboxydotrophic conditions rather than under organotrophic conditions (yeast extract-peptone-sulfur) [29]. A previous comparative study on the soluble proteome of *T. onnurineus* NA1 provided the first comprehensive view of all the metabolic pathways utilized by *T. onnurineus* NA1 when grown with three different substrate culture conditions that lead to H_2_ production [30]. As part of an effort to advance the knowledge of the metabolic capability and regulatory mechanisms in Thermococcales, we present herein the first global survey of sulfur metabolism at the protein level in hyperthermophiles, using a combination of 1D-SDS PAGE together with label free analysis by nano-UPLC-MS^E^. The quantitative proteomic analysis of *T. onnurineus* NA1 grown in the presence and absence of S° may provide critical insight to discover and understand the underlying molecular mechanisms of sulfur metabolism, as well as the possible metabolic pathway to provide potential targets of manipulation for improving hydrogen production. This report also gives basic insight into the metabolic adaptation of hyperthermophilic archaea in response to sulfur at the protein level, and provides a sound basis for further physiological and biochemical studies.

## 2. Results and Discussion

### 2.1. The Growth of T. onnurineus NA1 under Sulfur Culture Conditions

Previously, the growth and H_2_ production of *T. onnurineus* NA1 were characterized using various substrates under optimum growth conditions [14]. Growth on one-carbon substrates (CO, formate), peptone, and starch, as well as sulfur, was found to be supported by yeast extract. The ability of *T. onnurineus* NA1 to use elemental sulfur (S°) as an electron acceptor was also tested in cultures grown on YPS (yeast extract-peptone-sulfur) medium [14]. No growth was observed in the absence of yeast extract while utilizing sulfur as a growth substrate, which was most likely due to lack of biosynthetic pathways for several amino acids [15]. The optimal concentration of yeast extract to satisfy the growth requirements of *T. onnurineus* NA1 was found to be 3 g/L, but only when excess amounts of sulfur and peptone were provided, thereby defining the culture conditions for the proteomic experiments carried out herein. Growth in YPS medium resulted in the production of hydrogen sulfide (H_2_S), whereas H_2_ production was not detected [14]. Although sulfur was reduced to hydrogen sulfide, no negative effects of the presence of sulfur on cell density (specific growth rate) were observed. *T. onnurineus* NA1 reached higher cell density and growth when grown with S°, similar to what has been observed for *P. furiosus* [11]. These results indicate that *T. onnurineus* NA1 cells undergo a shift in cell metabolism for utilization of S° as an electron acceptor, switching the production of H_2_ to H_2_S. Therefore, it was expected that comparative analysis of the proteomic responses to sulfur and the three growth substrates (CO, formate, and starch) would enable the identification of a set of candidate proteins involved in sulfur metabolism, as well as the uncovering of a metabolic switch between sulfur reduction and H_2_ production in hyperthermophilic archaea.

### 2.2. Identification of Proteins Expressed under Sulfur Culture Conditions

To gain a clear picture of the processes and protein components involved in sulfur metabolism, the proteome of *T*. *onnurineus* NA1 cells grown on YPS medium containing S° was analyzed and compared with those of cells grown on H_2_-producing media containing formate, CO, or starch as growth substrates. A total of 589 proteins were identified from the peptide extract of each 1D-SDS/PAGE gel fraction using a nano-UPLC-MS^E^ method similar to that previously described [30]. The nano-UPLC-MS^E^ method, based on label-free quantitation, proved to be robust and allowed the quantitative comparison of all detectable proteins among the complex samples when separated by 1D PAGE [31,32]. A list of identified proteins along with quantitative data is provided in Supplementary Table S1. Quantitation of proteins expressed under particular growth conditions is also presented in Table 1. In total, 589 distinct proteins with more than two peptide fragmentations were identified at least twice across the experiment, which accounts for 29.8% of the 1976 genes predicted in the *T. onnurineus* NA1 genome. Of the 589 proteins, we categorized those whose expression was up-regulated ≥1.5 times or down-regulated ≤0.67 times with a *p* value <0.05 under the particular growth condition (Table 1). A total of 444 proteins for CO *versus* sulfur-grown cells, 445 for formate *versus* sulfur-grown cells, and 471 for starch *versus* sulfur-grown cells were selected. Using our criterion (≥1.5-fold change), 169, 159, and 143 proteins were found to be differentially abundant in CO *versus* sulfur-grown, formate *versus* sulfur-grown, and starch *versus* sulfur-grown cells, respectively. These differentially abundant proteins were considered to be candidates for having roles specific to growth with sulfur. To estimate abundance changes during growth on CO, formate, starch, and sulfur, and abundance ratio was also calculated for each protein (Table 1, Table 2 and Table S1). Proteins defined as up- or down-regulated, on the basis of whether peptide abundances were higher or lower in the sulfur-grown cells compared with the CO-, formate- and starch-grown cells (CO *versus* sulfur-grown, formate *versus* sulfur-grown, and starch *versus* sulfur-grown cells), respectively. We imposed abundance ratio to be greater than 1 (*p* value < 0.05, *p* value > 0.95; The confidence ≥ 95%) to consider a protein as sulfur-up-regulated. Proteins with ratio less than 1 (*p* value < 0.05, *p* value > 0.95; The confidence ≥ 95%) were considered as significantly down-regulated during growth on sulfur compared with other substrates. Thus, *T*. *onnurineus* NA1 cells may have evolved strategies that enable them to inhabit hot, sulfur-rich environments by regulating the major metabolic process through changes in protein abundance. The identified proteins were functionally categorized based on the KEGG database (Table S1). In an attempt to assign putative function, BLAST homology searches were performed. For cases in which significant homology was observed, a putative protein name and function were assigned. The identified proteins with significant homology to proteins annotated in *T. kodakarensis* KOD1 or *P. furiosus* were then classified into the same functional categories. The proteins fell into 18 major general functional categories that could be further divided into sub-function categories. Among the differentially detected proteins, some with unknown or poorly defined biological function were also present. Of the 589 proteins, 22.9% belonged to the “poorly characterized” category, annotated as hypothetical proteins or conserved hypothetical proteins. In total, 48 of the 135 hypothetical proteins were found to be up-regulated under sulfur culture conditions. These proteins may be important for the adaptation of *T*. *onnurineus* NA1 to survive in a sulfur-rich environment, and further research focusing on this group of proteins can not only help to deduce the response of *T*. *onnurineus* NA1 cells under sulfur culture conditions, but also provide a basis to formulate hypotheses about their biological functions.

**Table 1:** Summary of quantitative proteomic analysis of *Thermococcus onnurineus* NA1.

Substrate Ratio (A/B)	Sulfur/CO	Sulfur/Formate	Sulfur/Starch
*p* Value < 0.05 ^a^	330	336	359
A/B ≥ 1.5	169	159	143
A/B ≤ 0.67	130	141	172
1.5 > A/B > 0.67	31	36	44
*p* Value > 0.05	114	109	112
Subtotal	444	445	471
Total	–	589	–

^a^ Proteins exclusively identified depending on culture conditions were included in the protein group of *p* values <0.05.

### 2.3. General Trends in the Proteome of T. onnurineus NA1 Grown with S°

**Figure 1:**
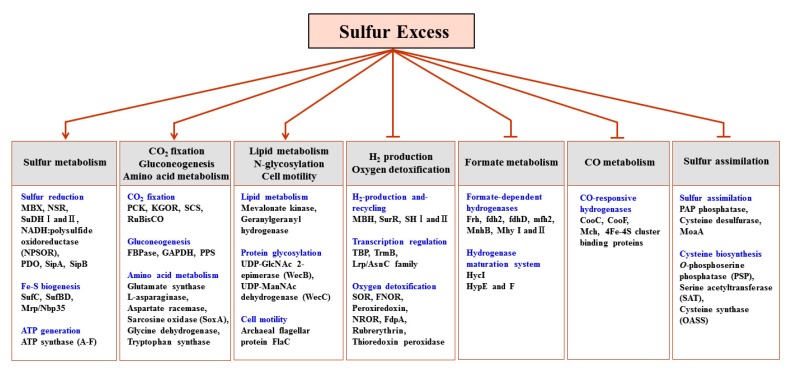
Functional categorization of the differentially expressed proteins as a function of the specific response to sulfur (Arrows indicates positive regulation, whereas bars indicate negative regulation).

To analyze whether the observed proteomic changes were specific for sulfur response, the protein expression profiles of the data collected under the four different substrate culture conditions were compared. Such comparative proteomics enabled the identification of a set of candidate proteins whose prominent presence was associated with the utilization of sulfur in response to cellular metabolic needs. The proteins identified in this manner could be sorted into 12 categories (Figure 1) according to their functional roles in response to sulfur growth conditions. The proteins within each functional category were divided into those up-regulated or down-regulated when *T. onnurineus* NA1 was grown with excess sulfur. The functional categories for all proteins observed are summarized in Table 2 and Table 3.

Clear trends in the protein expression of *T. onnurineus* NA1 cells grown on sulfur were observed. The data first revealed that, at the protein level, *T. onnurineus* NA1 displayed two antagonistic responses during sulfur addition. That is, the down-regulation of proteins involved in H_2_ metabolism, transcriptional regulation, and oxygen detoxification, and the up-regulation of proteins involved in S° reduction, Fe–S cluster biogenesis, ATP synthesis, CO_2_ fixation, protein glycosylation, lipid biosynthesis, proteolytic pathways, protein translocation, and formate oxidation to CO_2_ (Figure 1, Table 2 and Table 3). The up-regulation of an NADPH-dependent S° reducing system involving NAD(P)H:S° oxidoreductase (NSR) and membrane bound oxidoreductase (MBX) was similar to that proposed by the transcriptional analyses of *P. furiosus* [11], but the results obtained herein provide more detailed information regarding which metabolic enzymes were specifically induced during growth on excess sulfur.

**Table 2:** Relative abundance of representative up-regulated proteins in *T. onnurineus* NA1 cells grown on sulfur compared with those grown on CO, formate, and starch. (Y, only detected in Sulfur; S, only detected in Starch; C, only detected in CO; F, only detected in Formate).

Protein Name	Gene Identification	Ratio ^a^			*p* Value ^b^		
		Sulfur/CO	Sulfur/Formate	Sulfur/Starch	Sulfur/CO	Sulfur/Formate	Sulfur/Starch
gltB-1 Glutamate synthase small chain (SuDH I, SudA)	TON_0057	Y	Y	Y	Y	Y	Y
NADH:polysulfide oxidoreductase (NPSOR)	TON_0129	1.36	1.17	1.70	0.89	0.75	0.99
NADH oxidase (NSR)	TON_0305	Y	3.00	2.39	Y	1.00	1.00
Protein disulfide oxidoreductase (PDO)	TON_0319	2.53	3.06	1.40	1.00	1.00	1.00
ATPase *C*-terminus (SipB)	TON_0916	Y	Y	Y	Y	Y	Y
Iron-molybdenum cofactor-binding protein (SipA)	TON_0919	Y	Y	Y	Y	Y	Y
Putative oxidoreductase (SuDH II, SudX)	TON_1336	Y	Y	Y	Y	Y	Y
Ferredoxin-NADP^+^ reductase subunit α (SuDH II, SudY)	TON_1337	Y	Y	Y	Y	Y	Y
NADH dehydrogenase subunit D	TON_0487	2.48	Y	1.75	1.00	Y	0.97
V-type ATP synthase subunit E	TON_1749	Y	1.30	1.35	Y	0.65	0.77
V-type ATP synthase subunit C	TON_1750	Y	Y	1.36	Y	Y	0.69
V-type ATP synthase subunit F	TON_1751	Y	Y	Y	Y	Y	Y
V-type ATP synthase subunit A	TON_1752	2.12	1.57	1.79	1.00	1.00	1.00
V-type ATP synthase subunit B	TON_1753	2.66	1.58	1.88	1.00	1.00	1.00
V-type ATP synthase subunit D	TON_1754	Y	Y	Y	Y	Y	Y
ABC-type transport system involved in Fe–S cluster assembly, ATPase component (SufC)	TON_0530	Y	0.92	0.85	Y	0.40	0.41
ABC-type transport system involved in Fe–S cluster assembly (SufBD)	TON_0531	0.74	0.84	Y	0.27	0.35	Y
Hypothetical protein TON_0849 (SufBD-domain containing protein)	TON_0849	4.26	2.29	0.37	1.00	1.00	0.00
Hypothetical protein TON_0850 (SufBD-domain containing protein)	TON_0850	Y	Y	0.38	Y	Y	0.00
ATPase (MrP/Nbp35 family ATP-binding protein)	TON_1483	4.90	4.06	1.14	1.00	1.00	0.96
Phosphoenolpyruvate carboxykinase (PCK)	TON_0192	0.90	1.13	2.16	0.26	0.80	1.00
2-Oxoglutarate ferredoxin oxidoreductase subunit alpha (KGOR_α)	TON_0584	0.97	Y	Y	0.41	Y	Y
2-Oxoglutarate ferredoxin oxidoreductase subunit beta (KGOR_β)	TON_0586	Y	Y	1.58	Y	Y	0.69
Ribulose bisphosphate carboxylase (RuBisCO) type III	TON_1234	1.12	1.28	1.32	0.96	1.00	1.00
Archaeal succinyl-CoA synthetase forming), large subunit (SCS)	TON_1665	2.51	2.10	1.84	1.00	1.00	1.00
Phosphoenolpyruvate synthase (PPS)	TON_0311	1.77	1.68	0.96	1.00	1.00	0.11
Glyceraldehyde-3-phosphate dehydrogenase (GAPDH)	TON_0639	1.30	0.52	Y	0.89	0.00	Y
Thermophile-specific fructose-1,6-bisphosphatase (FBPase)	TON_1497	2.77	1.60	Y	1.00	1.00	Y
Mevalonate kinase (MVK)	TON_0133	Y	Y	Y	Y	Y	Y
Geranylgeranyl hydrogenase	TON_0316	1.51	1.92	0.81	1.00	1.00	0.03
UDP-*N*-acetylglucosamine 2-epimerase (WecB)	TON_0501	Y	Y	1.17	Y	Y	0.58
UDP-*N*-acetyl-d-mannosaminuronate dehydrogenase (WecC)	TON_0502	1.90	1.93	1.45	0.98	1.00	1.00
Archaeal flagella-related protein C (FlaC)	TON_1184	Y	Y	Y	Y	Y	Y
Bifunctional carboxypeptidase/aminoacylase	TON_0348	4.26	2.20	Y	1.00	0.93	Y
Methionine aminopeptidase	TON_0362	Y	Y	Y	Y	Y	Y
Deblocking aminopeptidase (DAP)	TON_0369	1.55	1.52	1.65	1.00	1.00	1.00
PepQ-1 X-pro dipeptidase	TON_0481	4.39	3.56	0.76	1.00	1.00	0.00
ATP-dependent protease Lon	TON_0529	Y	Y	Y	Y	Y	Y
Hypothetical endoglucanase	TON_0570	1.38	1.17	1.15	0.93	0.72	0.72
Prolyl endopeptidase	TON_0611	Y	Y	1.60	Y	Y	0.88
Xaa-Pro aminopeptidase	TON_0651	2.86	2.41	1.68	0.95	0.85	0.81
Zinc-dependent protease	TON_0804	Y	Y	0.58	Y	Y	0.23
Zinc-dependent protease	TON_0805	Y	Y	1.30	Y	Y	0.57
Acylamino acid-releasing enzyme (acylaminoacyl-peptidase)	TON_0969	Y	Y	Y	Y	Y	Y
Deblocking aminopeptidase (DAP)	TON_1032	2.83	2.23	1.75	1.00	1.00	1.00
d-aminopeptidase	TON_1067	Y	Y	Y	Y	Y	Y
Intracellular protease I (Pfp1)	TON_1285	Y	Y	1.55	Y	Y	0.99
Proteasome-activating nucleotidase (PAN)	TON_1385	Y	Y	Y	Y	Y	Y
Acylamino acid-releasing enzyme (acylaminoacyl-peptidase)	TON_1543	5.87	Y	Y	1.00	Y	Y
Cobalt-activating carboxypeptidase	TON_1687	26.05	8.33	3.22	1.00	1.00	1.00
d-aminopeptidase	TON_1960	Y	Y	Y	Y	Y	Y
Signal recognition particle protein Srp54	TON_0123	Y	Y	Y	Y	Y	Y
Signal recognition particle GTPase	TON_0592	Y	Y	1.42	Y	Y	0.77
Preprotein translocase subunit SecD	TON_1744	Y	Y	Y	Y	Y	Y
Prefoldin subunit α	TON_0914	Y	Y	Y	Y	Y	Y
Tryptophan synthase subunit β	TON_0147	Y	Y	Y	Y	Y	Y
Imidazolonepropionase-like amidohydrolase	TON_0704	2.77	4.22	1.97	1.00	1.00	1.00
Hypothetical aspartate racemase	TON_0801	Y	Y	Y	Y	Y	Y
l-asparaginase	TON_1392	Y	Y	Y	Y	Y	Y
Putative glutamate synthase subunit β	TON_0702	Y	Y	1.46	Y	Y	0.68
4-Aminobutyrate aminotransferase	TON_1605	Y	4.35	14.15	Y	1.00	1.00
Serine hydroxymethyltransferase	TON_0821	2.08	2.92	2.23	1.00	1.00	1.00
Sarcosine oxidase, α subunit	TON_1282	Y	Y	Y	Y	Y	Y
Glycine cleavage system protein H	TON_1334	2.23	Y	Y	0.97	Y	Y
Glycine dehydrogenase subunit 1	TON_0213	1.43	1.93	1.43	0.94	1.00	0.95
Glycine dehydrogenase subunit 2	TON_0214	1.63	1.86	1.82	1.00	1.00	1.00
l-Threonine 3-dehydrogenase	TON_0397	1.20	1.34	1.30	1.00	1.00	1.00
d-isomer specific 2-hydroxyacid dehydrogenase	TON_0569	1.77	1.72	1.34	0.99	1.00	0.92
*S*-adenosyl-l-homocysteine hydrolase	TON_1212	Y	1.40	Y	Y	0.80	Y
Diaminopimelate aminotransferase	TON_1785	2.2	1.32	Y	0.97	0.87	Y
50S ribosomal protein L4P	TON_0067	1.23	1.72	1.28	1.00	1.00	1.00
50S ribosomal protein L19e	TON_0084	1.12	Y	Y	0.60	Y	Y
50S ribosomal protein L18P	TON_0085	1.28	1.21	1.31	0.99	0.95	1.00
50S ribosomal protein L30P	TON_0087	1.46	1.62	1.51	1.00	1.00	0.98
50S ribosomal protein L15P	TON_0088	1.32	2.16	1.68	0.90	1.00	0.99
50S ribosomal protein L14e	TON_0094	1.43	1.92	1.75	1.00	0.99	0.99
Acidic ribosomal protein P0	TON_0181	1.15	1.55	1.42	0.98	1.00	1.00
50S ribosomal protein L21e	TON_0406	1.39	Y	2.23	0.85	Y	0.97
30S ribosomal protein S19P	TON_0070	Y	Y	Y	Y	Y	Y
30S ribosomal protein S4e	TON_0078	1.09	1.46	1.23	0.84	1.00	0.97
30S ribosomal protein S5P	TON_0086	1.11	1.34	1.26	0.82	0.98	0.99
30S ribosomal protein S13P	TON_0102	1.23	1.32	1.45	0.94	0.80	0.98
30S ribosomal protein S4	TON_0103	1.21	1.60	1.21	0.90	1.00	0.92
30S ribosomal protein S12P	TON_0222	1.02	1.67	1.27	0.52	0.98	0.96
Asparagine synthetase A	TON_0058	Y	Y	Y	Y	Y	Y
Leucyl-tRNA synthetase	TON_0141	Y	Y	1.26	Y	Y	0.78
Alanyl-tRNA synthetase-related protein	TON_0899	Y	Y	Y	Y	Y	Y
Isoleucyl-tRNA synthetase	TON_1803	1.82	1.51	2.80	1.00	0.99	1.00
Elongation factor 1-β	TON_1866	Y	Y	Y	Y	Y	Y
Translation initiation factor IF-2 subunit γ	TON_1944	1.31	1.09	1.31	0.99	0.79	1.00
Large helicase-related protein	TON_0613	Y	Y	Y	Y	Y	Y
Hypothetical phosphate transport system regulator PhoU	TON_1464	1.34	1.20	1.46	0.97	0.88	0.99
ABC transporter tungsten-binding protein (Tungsten)	TON_0014	Y	1.51	1.22	Y	0.88	0.79
Small-conductance mechanosensitive channel	TON_0799	Y	Y	Y	Y	Y	Y
ABC-type dipeptide/oligopeptide transport system (AppABC/OppBCDF)	TON_1764	8.50	5.21	3.32	1.00	1.00	1.00
ABC-type dipeptide/oligopeptide transport system, ATPase component	TON_1767	Y	Y	2.29	Y	Y	0.92
ABC-type dipeptide/oligopeptide transport system, ATPase component (AppABC/OppBCDF)	TON_1768	4.06	2.64	3.71	0.99	0.99	1.00
ABC transporter related ATPase component	TON_1874	Y	Y	Y	Y	Y	Y
Hydrogenase 4, component G or formate hydrogen lyase, subunit 5 (Mhy I)	TON_0276	1.73	Y	2.94	0.94	Y	0.94
Oxidoreductase iron-sulfur protein (Fdh1B)	TON_0280	Y	Y	Y	Y	Y	Y
fdhA formate dehydrogenase, α subunit (FdhA)	TON_0281	Y	Y	Y	Y	Y	Y

^a^ Ratio measured by MS^E^ methods; ^b^ *p* values calculated from individual peak intensities derived from all tryptic peptides detected per protein (see Experimental Section).

**Table 3:** Relative abundance of representative down-regulated proteins in *T. onnurineus* NA1 cells grown on sulfur compared with those grown on CO, formate, and starch (Y, only detected in Sulfur; S, only detected in Starch; C, only detected in CO; F, only detected in Formate; -, none detected in this experiment.).

Protein Name	Gene Identification	Ratio ^a^			*p* Value ^b^		
		Sulfur/CO	Sulfur/Formate	Sulfur/Starch	Sulfur/CO	Sulfur/Formate	Sulfur/Starch
Hypothetical transcription regulator (SurR)	TON_0318	-	-	S	-	-	S
Membrane bound hydrogenase, NiFe-hydrogenase large subunit 2	TON_1593	C	-	S	C	-	S
Cytochrome-c3 hydrogenase subunit gamma (SH II, Sulf-II)	TON_0054	-	-	S	-	-	S
Cytosolic NiFe-hydrogenase, α subunit (SH I, Sulf-I)	TON_0534	0.32	-	0.57	0.00	-	0.11
Cytosolic NiFe-hydrogenase, δ subunit (SH I, Sulf-I)	TON_0535	C	-	-	C	-	-
Cytochrome-c3 hydrogenase subunit gamma (SH I, Sulf-I)	TON_0536	C	-	-	C	-	-
Sulfhydrogenase beta subunit (SH Iβ)	TON_0537	C	-	-	C	-	-
ATPase involved in chromosome partitioning	TON_0262	0.78	0.53	0.47	0.07	0.00	0.00
Hydrogenase maturation protease HycI	TON_0263	-	F	-	-	F	-
Formate hydrogen lyase, subunit 7 (Mhy I)	TON_0274	-	-	S	-	-	S
Hydrogenase maturation protein HypF	TON_0286	0.26	0.41	0.18	0.01	0.06	0.00
Hydrogenase expression/formation protein HypE	TON_0287	0.69	0.86	0.45	0.16	0.41	0.00
Coenzyme F420 hydrogenase alpha subunit (Frh_α)	TON_1559	-	F	S	-	F	S
CoenzymeF420 hydrogenase/dehydrogenase beta subunit (Frh_β)	TON_1561	-	F	S	-	F	S
TonB-dependent receptor protein:Formate dehydrogenase, subunit FdhD	TON_1562	-	F	-	-	F	-
Hypothetical formate dehydrogenase, α subunit (Fdh2)	TON_1563	C	F	S	C	F	S
4Fe-4S cluster-binding protein	TON_1564	-	F	-	-	F	-
Hydrogenase 4, component G or formate hydrogen lyase, subunit 5 (Mhy II, Mfh2)	TON_1569	-	F	-	-	F	-
Formate hydrogen lyase subunit 6 (Mhy II)	TON_1570	-	F	-	-	F	-
Hydrogenase 4, component I or formate hydrogen lyase, subunit 7	TON_1571	-	F	-	-	F	-
Hypothetical protein TON_1572	TON_1572	-	F	-	-	F	-
Hypothetical Multisubunit Na^+^/H^+^ antiporter MnhB subunit (MnhB)	TON_1577	-	F	-	-	F	-
NADH dehydrogenase subunit C (MBX)	TON_0488	C	-	-	C	-	-
4Fe-4S ferredoxin, iron-sulfur binding domain protein (CooF)	TON_1017	C	-	-	C	-	-
Hypothetical ATP-binding protein (CooC)	TON_1019	C	-	-	C	-	-
Hydrogenase 4, subunit 5 (Mch)	TON_1023	C	-	-	C	-	-
NADH dehydrogenase (ubiquinone), 20 kDa subunit (Mch)	TON_1024	C	-	-	C	-	-
Transcription regulator, PadR family	TON_0114	-	F	-	-	F	-
Hypothetical transcription regulator (TrmB)	TON_0332	0.76	0.81	0.54	0.00	0.00	0.00
Hypothetical transcription regulator (Lrp/AsnC family transcriptional regulator)	TON_0662	-	F	-	-	F	-
Transcription regulator (Lrp/AsnC family transcriptional regulator)	TON_1284	-	F	S	-	F	S
Transcription factor (TBP)	TON_1309	-	F	S	-	F	S
Transcription regulator (Phosphate uptake regulator, phoU)	TON_1393	-	-	S	-	-	S
Hypothetical transcription regulator	TON_1436	-	-	S	-	-	S
Transcription regulator (Lrp/AsnC family Transcription regulator)	TON_1510	0.39	0.29	0.54	0.00	0.00	0.02
Transcription regulator, ArsR family	TON_1663	-	-	S	-	-	S
Hypothetical transcription regulator (TrmB-like protein)	TON_1797	0.82	0.73	0.44	0.35	0.19	0.00
Manganese-dependent transcription regulator	TON_1956	-	-	S	-	-	S
Peroxiredoxin	TON_0829	0.17	0.08	0.16	0.00	0.00	0.00
Thioredoxin peroxidase	TON_0862	C	-	S	C	-	S
Type A flavoprotein (FdpA, flavodiiron protein)	TON_0863	0.36	0.41	0.46	0.00	0.00	0.00
NAD(P)H:rubredoxin oxidoreductase (NROR)	TON_0865	C	-	S	C	-	S
Rubrerythrin (Rr)	TON_0866	0.34	0.48	0.70	0.00	0.00	0.00
Sor superoxide reductase (SOR)	TON_0868	-	0.32	-	-	0.00	-
Ferredoxin-NADP^+^ reductase subunit α (FNOR)	TON_0056	-	-	S	-	-	S
Cysteine desulfurase	TON_0289	C	F	S	C	F	S
Cysteine synthase (OASS)	TON_1004	-	-	S	-	-	S
Hypothetical protein TON_1360 (SAT, serine acetyltransferase)	TON_1360	-	-	0.88	-	-	0.44
Molybdenum cofactor biosynthesis protein A (MoaA)	TON_1410	-	F	S	-	F	S
Hypothetical protein TON_1504 (PSP, *O*-phosphoserine phosphatase)	TON_1504	0.66	0.41	0.53	0.18	0.03	0.09
Hypothetical protein TON_1706 (PAP phosphatase)	TON_1706	-	F	-	-	F	-

^a^ Ratio measured by MS^E^ methods; ^b^ *p* values calculated from individual peak intensities derived from all tryptic peptides detected per protein (see Experimental Section).

### 2.4. Proteins Up-Regulated in Response to Growth on Sulfur

#### 2.4.1. CO_2_ Fixation

The signature feature of the proteome of cells grown on S° was up-regulation of proteins involved in carbon fixation, namely, ribulose 1,5 bisphosphate (RuBP) carboxylase/oxygenase type III (RuBisCO: TON_1234), phosphoenolpyruvate carboxykinase (PCK: TON_0192), 2-oxoglutarate: ferredoxin oxidoreductase subunit (KGOR: TON_0584, and TON_0586), and succinyl-CoA synthetase large subunit (SCS: TON_1665), as shown in Figure 1 and Table 2. Similar to the results reported in the genomic analyses of other Thermococcales [23,24], *T. onnurineus* NA1 contains the enzymes necessary for a pseudo-TCA cycle [30]. Although ATP-dependent citrate lyase has not yet been detected in the *T. onnurineus* NA1 proteome, the up-regulation of RuBisCO (TON_1234), 2-oxoglutarate:ferredoxin oxidoreductase activities (KGOR: TON_0583-0588), phosphoenolpyruvate carboxykinase (PCK), and other CO_2_ fixation enzymes in CO- or sulfur- grown *T. onnurineus* cultures led to the suggestion that this organism can fix CO_2_ via the reductive TCA cycle. Under CO culture condition**,** our previous proteomic analysis showed significant up-regulation of these enzymes involved in CO-dependent anabolic pathway for assimilation of CO_2_ via a reductive TCA cycle [30]. In addition, our previous data suggest that pyruvate formed from acetyl-CoA and CO_2_ by the reverse reaction of pyruvate ferredoxin oxidoreductase (POR) can enter the CO-dependent anabolic pathway, generating cellular carbon via the reductive TCA cycle [30]. This CO-dependent anabolic flux appears to be important for *T. onnurineus* NA1, because it could assimilate CO_2_ into celluar carbon via the rTCA cycle. In the present study, RuBisCO type III (TON_1234) was strongly up-regulated in cultures grown on sulfur compared to those grown on H_2_-evolving substrates. Intriguingly, Type III RuBisCO from *T. kodakarensis* KOD1 has been shown to complement a RuBisCO-deficient strain of the purple non-sulfur bacterium *Rhodopseudomonas palustris*, and therefore, is able to operate in Calvin-Benson-Bassham (CBB) pathway mode [33]. It has also been proposed that type III RuBisCO functions in a novel AMP recycling pathway [34]. Ribose 1,5-bisphosphate isomerase (RBPI) and AMP phosphorylase (DeoA), enzymes required to supply the RuBisCO substrate, are both present in the *T. onnurineus* NA1 genome [15]. As shown in Supplementary Table S1, RBPI homolog (TON_1296) and AMP phosphorylase (DeoA, TON_1062), two major enzymes of AMP recycling pathway, were also detected in our proteomic analysis. However, RBPI (TON_1296) was up-regulated in cells grown on sulfur, and the induction level of DeoA (TON_1062) was slightly low in sulfur-grown culture compared to CO-grown culture. Interestingly, our previous transcriptomic analysis showed that gene expression of these enzymes was up-regulated in sulfur–grown cells compared with CO-grown cells (data not shown). Therefore, under sulfur culture conditions, the most likely physiological role of RuBisCO type III in *T. onnurineus* NA1 is in carbon fixation, as well as in the AMP recycling pathway. In other words, these results suggest that RuBisCO type III (TON_1234) catalyzes the assimilation of CO_2_ to cellular carbon during growth on sulfur. It was also verified by our previous studies that mRNA expression of enzymes involved in CO_2_ fixation including RuBisCO (TON_1234) was up-regulated in cells grown on sulfur compared to CO (data not shown). In a previous study with *T. onnurineus* NA1, the expression of a selected group of genes was investigated using a whole-genome DNA microarray by comparing mRNA expression from cells grown on sulfur with CO [19]. On average the proteomic and transcriptomic data correlate reasonably well.

In addition, enzymes involved in gluconeogenesis (GAPDH: TON_0639, FBPase: TON_1497, PPS: TON_0311), except for PGK (TON_0742), were specifically induced during growth on sulfur. In fact, PGK (TON_0742) was detected with higher abundance in cells grown with formate than with other substrates, as recently revealed in our previous proteomic study [30]. Although these proteins are soluble proteins that are primarily in formate-grown cells, *T. onnurineus* NA1 also responded to sulfur stress by up-regulating some gluconeogenic enzymes. The reasons for up-regulation of the proteins related to CO_2_ fixation remain unclear. However, these up-regulated proteins are presumed to function in the provision of intracellular fixed carbon via the reductive TCA (rTCA) cycle. *T. onnurineus* NA1 is a sulfur-reducing anaerobe that prefers amino acids as carbon and energy sources in the presence of sulfur [13,28]. Sulfur reduction requires the formation of reduced electron carriers, such as NADPH and reduced ferredoxin (Fd_red_). In *P. furiosus* and related heterotrophic Thermococcales, abundant NADPH can be generated from the oxidative deamination of amino acids by the concerted action of glutamate dehydrogenase (GDH) and aminotransferase (AT) [6,35]. Excess NADPH is proposed to be recycled through the reduction of sulfur to hydrogen sulfide. In addition, the reduction of sulfur also creates a proton motive force for ATP production [12]. The generated ATP, NADPH, and reduced ferredoxin are then used to drive the rTCA cycle for metabolism, and the energy-demanding CO_2_ fixation reaction. Thus, FADH_2_ and NADPH fluxes into biomass and the rTCA cycle are stronger in cultures grown on sulfur, consequently supporting a higher growth rate during heterotrophic growth with sulfur. As recently described in other Thermococcales [23,24], the CO_2_ produced from amino acid catabolism or CO oxidation may be a suitable substrate for the type III RuBisCO (TON_1234), which together with DeoA (TON_1062) and RBPI (TON_1296) replenish the 3-phosphoglycerate used in gluconeogenesis. Therefore, it may potentially be metabolically favorable to fix CO_2_ and channel some of the fixed carbon into carbohydrate synthesis when hydrogen sulfide is available as an energy source in environments with limited organic carbon, such as around hydrothermal vents.

#### 2.4.2. Lipid Biosynthesis

In the present study, mevalonate kinase (TON_0133), as well as geranylgeranyl hydrogenase (geranylgeranyl reductase: TON_0316), were found to be abundantly expressed during growth on sulfur (Figure 1 and Table 2). Assuming that mevalonate kinase is essential for the growth of *T. onnurineus* NA1 under all substrate culture conditions, the reasons for its apparent up-regulation during growth on sulfur compared with other substrates remain unexplained. Mevalonate kinase was demonstrated to be a key enzyme in the mevalonate pathway for the biosynthesis of the essential isoprenoid precursor, isopentenyldiphosphate (IPP) [36,37]. Geranylgeranyl hydrogenase, the enzyme responsible for the reduction of isoprenoid side chains in archaeal lipid biosynthesis, has been recently described in *Thermoplasma acidophilum* [38,39], *Archaeoglobus fulgidus* [40], and *Sulfolobus acidocaldarius* [41]. The reduction of isoprenoid side chains in archaeal lipid is generally thought to play an important role in the survival of archaea in extreme environments [42]. Moreover, saturation of isoprenoid side chains would confer chemical stability on archaeal membrane lipids [40]. Thus, *T. onnurineus* NA1 may have adapted by changing its membrane lipid composition in order to survive in environments with extremely high sulfur concentrations.

#### 2.4.3. Protein Glycosylation and Motility

Enzymes involved in protein glycosylation (TON_0501 and TON_0502) and flagella accessory protein FlaC (TON_1184) were also significantly up-regulated in cultures grown with sulfur (Figure 1 and Table 2). TON_0501 and TON_0502 are predicted to encode WecB (UDP-GlcNAc 2-epimerase) and WecC (UDP-*N*-acetyl-d-mannosamine dehydrogenase), which were found to be involved in the biosynthesis of UDP-acetamido sugars [43]*.* In most methanogens, halobacteria and Thermococcales, UDP-acetamido sugars are considered to be precursors for *N*-linked glycosylation of flagellin and S-layer proteins [44,45], and are involved in the modification of cytoplasmic factors such as folate and methanogenic coenzyme B [46,47,48]. Such protein glycosylation is believed to be important for the adaptation of archaea to the extreme environments in which these organisms can thrive [49,50]. Although its specific function has yet to be determined, N-glycosylation may be necessary to stabilize the proteins of *T. onnurineus* NA1 to cope with hot, sulfur-rich environments. More unexpectedly, the up-regulation of FlaC (TON_1184), which is part of the flagella operon, implies the importance of motility in cells cultured on sulfur, which would provide the ability to attach to the elemental sulfur, as observed in *Thiobacillus ferrooxidans* [51]. It was previously shown that *Thiobacillus ferrooxidans* cells could adhere to elemental sulfur through a disulfide bond with the 40-kDa protein in the flagella, thereby utilizing insoluble sulfur in their envelope.

#### 2.4.4. Proteolytic Pathways and Protein Translocation

In the presence of S°, *T. onnurineus* NA1 mainly used peptides as a carbon source, converting them into carboxylic acids, which is in accordance with the results previously reported for growth studies using *P. furiosus* [35]. As expected, a large set of enzymes involved in proteolytic pathways, protein export, protein folding, and amino acid metabolism were highly up-regulated in the cells grown with sulfur (Table 2). The up-regulation of several proteases was consistent with the previously reported proteomic and biochemical analyses, for which two deblocking aminopeptidases (DAPs; TON_0369 and TON_1032) were found in greater abundance in YPS-cultured cells [28,52]. In addition, the proteomic analysis herein found significant up-regulation of Lon protease (Lon, TON_0529) and proteasome-activating nucleotidase (PAN, TON_1385) in the sulfur-cultured cells (Table 2). The Lon protease (*Ton*Lon, TON_0529) of *T. onnurineus* NA1 has high sequence homology with a previously characterized LonB protease (*Tk*Lon) from *T. kodakarensis* KOD1 [53], for which the crystal structure was recently determined [54]. Together with the cytosolic PAN protease, archaeal LonB plays a key role in all ATP-dependent proteolysis for protein quality control and metabolic regulation [54,55].

#### 2.4.5. Formate Oxidation to CO_2_

Finally, the proteome data obtained herein also provided intriguing information on another class of enzymes, the hydrogenases (Table 2). To our surprise, formate hydrogen lyase Mhy I (TON_0276), formate dehydrogenase I subunit B (Fdh1B, TON_0280), and formate dehydrogenase (FdhA, TON_0281) were all found to be significantly up-regulated in sulfur-cultured cells, in contrast to the other hydrogenase cluster proteins which were down-regulated in the presence of sulfur. Presumably, the fdhA-MhyI complex is responsible for the removal of formate from the sulfur-cultured cells, which is likely generated during amino acid catabolism, by catalyzing the oxidation of formate to CO_2_, as proposed in *Thermococcus litoralis* [56].

#### 2.4.6. Reductive Sulfur Metabolism

**Figure 2:**
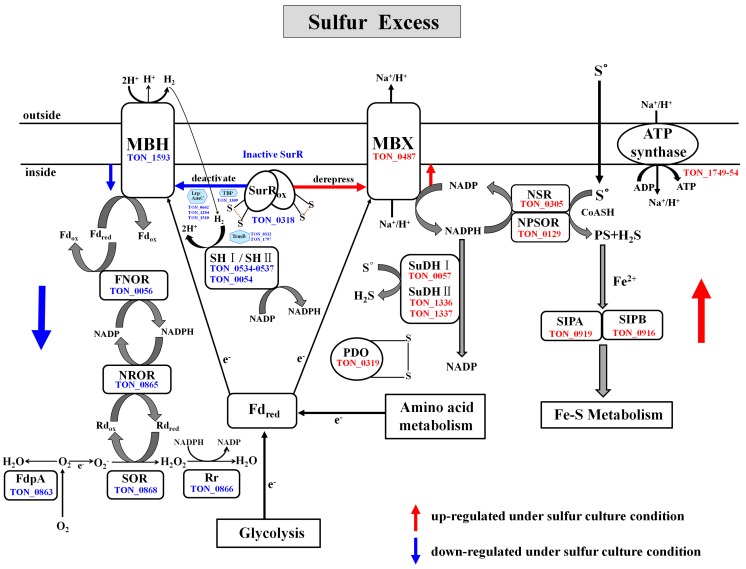
Pathways proposed for the reductive sulfur metabolism, H_2_-metabolism, and oxidative stress defense in *T*. *onnurineus* NA1. SurR is a redox-sensitive transcription regulator, which is down-regulated in the presence of S° via protein oxidation. When S° became available, oxidized SurR down-regulated the expression of the Mbh, TBP, TrmB, and Lrp/AsnC family of transcriptional regulators and the key enzymes involved in H_2_-metabolism, and oxygen detoxification, while concomitantly derepressing the expression of proteins related to S° reduction, such as MBX, as well as NSR, NPSOR and SuDH. All components depicted in the illustration were identified in comparative proteome analyses, and are represented by the name and gene identification numbers of *T*. *onnurineus* NA1. Down or up-regulated proteins (enzymes) during growth on sulfur are indicated with blue or red arrows, respectively.

Proteomic analysis identified a suite of genes that function in sulfur reduction, for which the protein products were more abundant in sulfur-cultured cells (Figure 2 and Table 2). Previously, a novel S°-reducing system involving NAD(P)H sulfur oxidoreductase (NSR, PF1186) and a membrane bound oxidoreductase (MBX, PF1441-PF1453) was reported in the hyperthermophilic archaeon *P. furiosus* [11,57]. NSR and MBX are proposed to be the key enzymes responsible for the reoxidation of ferredoxin and NAD(P)H [12]. In *T. onnurineus* NA1, NSR (TON_0305), a homolog of PF1186, was found to be highly up-regulated under sulfur culture conditions. The *T. onnurineus* NA1 genome contains gene clusters which are predicted to encode the 13 subunits of MBX (TON_0486-TON_0498). The *Mbx* gene clusters of *T. onnurineus* NA1 are arranged identical to those (PF1441-PF1453) in *P. furiosus,* reported to comprise an operon, and were found to be transcriptionally up-regulated in the S°-grown cells [11]. In previous proteomic analysis of *T. onnurineus* NA1, most of the MBX subunits (TON_0486-TON_0498), except for TON_0495, were shown to be expressed during growth on S°-containing YPS medium [29]. In the present study*,* only two MBX subunits were detected. One (TON_0487) was up-regulated in cells grown with S°, while the other (TON_0488) was down-regulated (and up-regulated in CO-grown cells). It is intriguing to note that the observed up-regulation of one MBX subunit (TON_0487) is in agreement with the results of transcriptional analysis on *P. furiosus,* while expression of the other (TON_0488) showed a discrepancy. It is not clear why TON_0488, one of the MBX subunits that are expressed in clusters, appears to be down-regulated in the *T. onnurineus* NA1 cells grown with S°. However, this difference might be caused by post-transcriptional regulation of *Mbx* gene expression, occurring in response to sulfur.

Unlike in *P. furiosus*, NADH: Polysulfide oxidoreductase (NPSOR: TON_0129), a close homolog of *T. kodakarensis* KOD1 NAD(P)H oxidase (NOX: TK1481) that is capable of generating H_2_S [23,58], was up-regulated in the comparative proteome for cells grown on sulfur. In the presence of sulfur, MBX oxidized the reduced ferredoxin, and is proposed to transfer electrons to NAD(P) for generation of NAD(P)H, which is subsequently used by the sulfur reducing enzyme NSR for the conversion of S° to H_2_S [12]. Although NPSOR (TON_0129) most likely functions in sulfur reduction, it may also produce sulfide from polysulfide in the cytoplasm for assimilation, as proposed in the thermophilic, sulfur-reducing epsilonproteobacterium *Nautilia profundicola* [59]. In *T. onnurineus* NA1, little formation of polysulfides may be obtained by the reaction of S° with sulfide, which readily occurs at hydrothermal vents and is also found in the culture medium. MBX exports protons to create an ion-gradient across the membrane that is used in the synthesis of ATP by ATP synthase [11,12]. Specifically, six subunits of vacuolar (V)-type ATP synthase, namely A, B, C, D, E and F, were expressed at higher levels during growth on sulfur (Table 2). As shown in Figure 2 and Table 2, the protein disulfide oxidodreductase (PDO: TON_0319), a SurR-regulated glutaredoxin-like protein proposed to be involved in the maintenance of protein disulfide bonds [60], was also strongly up-regulated during growth on sulfur, although it’s specific role in S° metabolism has yet to be defined. *T. onnurineus* NA1 also contains sulfide dehydrogenase I (SuDH I, SudA: TON_0057) and II (SuDH II:Sud XY, TON_1336-1337), which are homologous to the *P. furiosus* enzymes [12,61,62] and were strongly up-regulated during growth on sulfur. Report of a similar pattern of transcriptional up-regulation for the SuDH II (PF1910-PF1911, *sudXY*) of *P. furiosus* [63] suggests that these enzymes serve an important function in the NADPH-dependent reduction of S°. Recently, it was proposed that two SuDH isozymes from *P. furiosus* can compensate for the absence of NSR in S° reduction [12]*.*

#### 2.4.7. Fe–S Cluster Biogenesis

In a previous study of *P. furiosus*, PF2025 was found to be the most significantly up-regulated gene when grown in the presence of sulfur, along with another ORF, PF2026 [10,11]. Their products, renamed SipA and SipB (for sulfur-induced proteins), respectively, and gene deletion study also suggested that they may be involved in iron-sulfur metabolism [12]. In the present study, the levels of SipA (TON_0919) and SipB (TON_0916) proteins increased significantly under sulfur culture conditions. In addition, several proteins involved in iron-sulfur cluster biogenesis were also found to be up-regulated to some extent, including SufC (TON_0530), SufBD-related proteins (TON_0531 and TON_0849-0850), and Fe–S cluster carrier protein Mrp/Nbp35 family ATP-binding protein (TON_1843). Such archaeal Suf proteins constitute the iron sulfur cluster assembly machinery [2]. These results indicate that *T. onnurineus* NA1, like the other archaea *P. furiosus* and *Methanococcus maripaludis* [12,64], may be able to utilize sulfide as a sulfur source for iron-sulfur cluster biogenesis under S°-reducing conditions.

### 2.5. Antagonistic Switch between H_2_-Metabolism and Reductive Sulfur Metabolism

Under sulfur culture conditions, our comparative proteome data showed exactly the opposite regulation, with hydrogenases-related proteins and several O_2_ detoxifying enzymes being down-regulated, and concomitant up-regulation of S°-responsive proteins. When S° becomes available, their respective expressions appears to be correlated, at least in part*,* with the control of the oxidized SurR which functions as a repressor of MBH and other hydrogenase-related proteins, and as a derepressor of MBX and S°-responsive proteins (Figure 2, Table 2 and Table 3). Consequently, a rapid switch from H_2_ production to S° metabolism occurs. Thus, our proteomic results support the previous transcriptional and genomic analysis data regarding the SurR-dependent on/off switch mechanism for the production of H_2_ or H_2_S [5,11,12].

### 2.6. Proteins Down-Regulated in Response to Growth on Sulfur

#### 2.6.1. H_2_-Metabolism

Under sulfur culture conditions, the key transcriptional regulator SurR (TON_0318), which activates the expression of hydrogenase genes in the absence of sulfur (S°) [65,66], was found to be down-regulated, along with MBH (TON_1593), which is involved in reoxidizing reduced ferredoxin (Fd_red_) to produce H_2_ [67] (Figure 2 and Table 3). *T. onnurineus* NA1 also contains two gene clusters (TON_0534 to 0537 and TON_0052 to 0055) which encode cytoplasmic hydrogenases homologous to SH I and SH II of *P. furiosus*, which have been predicted to be involved in H_2_ recycling to provide NADPH for biosynthesis [68,69]. As shown in Figure 2 and Table 3, two cytoplasmic [Ni–Fe] hydrogenases, SH I (Sulf-I: TON_0534 to 0537) and SH II (Sulf-II: TON_0054), were markedly down-regulated in cultures grown in the presence of sulfur, which is consistent with the reduced expression of the related genes in *P. furiosus* [8,11,35]. While *P. furiosus* and *T. onnurineus* NA1 both possess these two cytoplasmic [Ni–Fe] hydrogenases, only one homologue of SH I (Hyh-I) was previously identified in *T*. *kodakarensis* KOD1, which was found to be an H_2_ uptake hydrogenase acting *in vivo* during H_2_ production [6].

The response of *T. onnurineus* NA1 to S° is closely related with the decrease of its ability to produce hydrogen. Two major hydrogenase gene clusters which are known to be essential for H_2_ production and growth on formate and CO [15,16,17,19], belonging to Hyg4-III (fdh2-mfh2-mnh2: TON-1559-1582) and Hyg4-II (codH-mch-mnh3:TON_1016-1031), were found to be the most highly down regulated in the sulfur-cultured cells (Table 3). Noticeably, TON_1569 and TON_1023, which encode the large subunits mfh2 and mch hydrogenase, respectively, were previously identified to be prominently down-regulated in the validation of microarray data by reverse transcription-PCR (RT-PCR) analysis of RNA from cells grown on YPS medium [19]. Other Hyg4-III hydrogenases that were down-regulated under sulfur culture conditions included the F420 hydrogenases, Frh (TON_1559 and TON_1561) and formate dehydrogenase, FdhD (TON_1562). These protein complexes (FdhD and Frh), known as energy-conserving formate hydrogen lyase systems, convert formate into CO_2_ and H_2_ [16,30]. Besides the strong down-regulation of CO- and formate-responsive hydrogenase gene clusters mentioned above, known auxiliary proteins involved in hydrogenase maturation, including Hyc I (TON_0263), Hyp F (TON_0286), and Hyp E (TON_0287), belonging to the Hyg4-I (fdh1-mfh1-mnh1: TON_0261-0289) cluster [70,71], were also found to be significantly down-regulated during growth on sulfur. These proteome results are consistent with genetic studies of *P. furiosus,* which revealed the hydrogenase operons to be regulatory targets of SurR, along with genes involved in hydrogenase maturation whose expression is most highly down-regulated in the primary response to S° [65,66].

#### 2.6.2. Oxygen Detoxification

The *T*. *onnurineus* NA1 proteome also indicated the presence of a superoxide-reducing system for the detoxification of reactive oxygen species (ROS). Superoxide reductase (SOR: TON_0868), rubrerythrin (Rr: TON_0866), NAD(P)H:rubredoxin oxidoreductase (NROR: TON_0865), and flavodiiron proteins (FdpA: TON_0863), along with MBH and cytoplasmic hydrogenase SH I and SH II, were among the most strongly down-regulated proteins identified in the proteomic study herein. This finding was not observed in previous genomic studies of *P. furiosus*, although a deletion mutant lacking SOR and FdpA showed increased sensitivity to O_2_ and decreased H_2_ production, while there was no significant decrease in the activities of hydrogenases for H_2_ evolution and recycling when the cells were exposed to O_2_. Therefore, it has been proposed that electrons from sugar fermentation normally used to generate H_2_ via MBH are also diverted to O_2_ detoxification when the cells are exposed to O_2_ stress [72]. In the *T*. *onnurineus* NA1 proteome, ferredoxin:NAD(P) oxidoreductase (FNOR: TON_0056), a homolog to *Pyrococcus* COR I (PF1328), was also strongly down-regulated under sulfur culture conditions (Figure 2 and Table 3). In *P. furiosus*, cytoplasmic oxidoreductase I (COR I) is proposed to play a critical role in the connection of the reduced ferredoxin and NADPH pools [72], although the characteristics of this enzyme have not yet been described. Hence, the expression of enzymes involved in oxygen detoxification appears to be closely related to the production of H_2_, which is coupled with the redox reaction of ferredoxin. Reduced ferredoxin and NADP^+^ are converted to oxidized ferredoxin and NAD(P)H by this cytoplasmic oxidoreductase, and the NAD(P)H produced can also be used by NROR to reduce Rubredoxin (Rd) in the O_2_ reducing electron transfer chain.

#### 2.6.3. Transcription Regulation

Interestingly, a homolog of TBP (TON_1309) and some of its associated proteins, such as the Lrp/AsnC family of transcriptional regulators TON_0662, TON_1284, and TON_1510, as well as TrmB (TON_0332 and TON_1797), were differentially down-regulated in the proteome in response to sulfur. It was previously proposed for *P. furiosus* that some of these transcriptional regulators are closely related to the mechanism of activation or repression controlled by SurR [65]. TrmB is known to be the transcription repressor of the gene clusters encoding the trehalose/maltose ABC transporter in the hyperthermophilic archaea *T. litoralis* and *P. furiosus* [73,74]. The down-regulation of TrmB in sulfur-cultured cells suggests that inactivation of TrmB de-represses the expression of the trehalose/maltose transport operon and synthesis of the MALEFG-trehalose synthase. Due to the insufficient flow of glucose in the presence of sulfur, *T. onnurineus* NA1 may have the need to exert more effort by enhancing the expression of genes encoding for the uptake and synthesis of sugars. Based on the comparative proteomic results, the down-regulation of TrmB appeared to be parallel to the up-regulation of proteins involved in CO_2_ fixation and gluconeogenesis under sulfur culture conditions.

#### 2.6.4. Sulfur Assimilation Pathway

For biosynthesis, most organisms need to either obtain sulfur-containing amino acids (e.g., cysteine and methionine) from their environment, or synthesize them *de novo* by generating and capturing sulfide. Similar to many bacteria, *T. onnurineus* NA1 has also been shown to have a reductive sulfate assimilation pathway for production of sulfide in the cytoplasm [75]. *T. onnurineus* NA1 contains genes corresponding to APS kinase (TON_1704), pyrophosphatase (TON_1705), PAP phosphatase (TON_1706), and ATP sulfurylase (TON _1707), which are part of the proposed sulfur assimilation pathway involved in sulfite reduction and the formation of sulfur metabolites [75]. As shown in Supplementary Figure S1 and Table 3, PAP phosphatase (TON_1706) was specifically down-regulated in sulfur-cultured cells. In contrast to the marked decrease in PAP phosphatase (TON_1706) in the presence of sulfur, the expression of ATP sulfurylase (TON_1707) was noticeably increased, but was slightly less than in CO-grown cultures (Supplementary Table S1). In addition to the enzymes described above, other proteins required for sulfate assimilation, TON_0957 and TON_0360, which putatively encode PAPS reductase and sulfite reductase, respectively, were not found in the *T*. *onnurineus* NA1 proteome. Further study is needed to prove the functions of these enzymes in *T*. *onnurineus* NA1. The *T*. *onnurineus* NA1 genome also encodes two candidate proteins, TON_1958 and TON_1959, which share homology with putative sulfate transporters from *P. furiosus* (PF1519-1520 and PF1748-1750) [76]; However, no evidence of a functional sulfate transporter was detected in *T*. *onnurineus* NA1.

*O*-phosphoserine phosphatase (PSP, TON_1504), serine acetyltransferase (SAT, TON_1360), and *O*-acetylserine sulfhydrylase (OASS, TON_1004), which captures sulfide for cysteine and methionine synthesis, were also found to be down-regulated in the sulfur-cultured cells (Figure S1 and Table 3). These results indicate the possibility that *T. onnurineus* NA1 can utilize a bacterial cysteine biosynthesis pathway, as has been displayed by some *Methanosarcinales* species [2,77]. Cysteine is often present in the medium as a reductant. In hyperthermophilic archaea, cysteine fulfills the dual function of a reducing agent and a source of organic sulfur [78,79]. In the case of *T. kodakarensis* grown in the absence of S°, cysteine is thought to be an essential sulfur source for Fe-S cluster biogenesis via cysteine desulfurase activity [79]. In contrast to some methanogens and many other archaea from solfataric hydrothermal systems, the genome of *T*. *onnurineus* NA1 contains a homologue of cysteine desulfurase. It is important to note the down-regulation of cysteine desulfurase (TON_0289) in the sulfur-cultured *T*. *onnurineus* NA1 cells, as this enzyme has been shown to play a key role in the mobilization and transfer of sulfur from cysteine through an enzyme-bound persulfide intermediate to scaffold proteins [64]. Down regulation of cysteine desulfurase in *T*. *onnurineus* NA1 grown on sulfur is in agreement with a report wherein it was proposed that cysteine desulfurase activity is necessary for iron-sulfur cluster biogenesis in the absence of S° [79]. Furthermore, the *T*. *onnurineus* NA1 genome encodes a homolog of molybdenum cofactor biosynthesis protein (MoaA, TON_1410), the enzyme responsible for the incorporation of metal into the molybdopterin cofactor. Here, MoaA (TON_1410) was also observed to be remarkably down-regulated in the sulfur-cultured cells. Under S°-sufficient culture conditions, the down-regulation of enzymes involved in the assimilation of sulfate into cysteine and methionine suggest that the sulfate uptake and sulfur assimilation activities of *T. onnurineus* NA1 are likely to be more repressed when there is increased abundance of intracellular sulfide and polysulfide (and/or sulphane sulfur). Importantly, the data furthermore suggest that, in the presence of S°, sulfide or polysulfide produced from the reduction of S° is also capable of serving as the major sulfur source for methionine, cofactors, and Fe–S cluster synthesis, instead of the cysteine assimilated from sulfate in the medium, which is supported by the biochemical observations in methanogenic archaeon *Methanococcus maripaludis* [64].

## 3. Experimental Section

### 3.1. Strain and Culture Conditions

*T. onnurineus* NA1 was cultured in yeast extract/peptone/sulfur (YPS) medium containing (g/L) NaCl (35.0), Na_2_SO_4_ (3.3), KCl (0.05), H_3_BO_3_ (0.02), MgCl_2_·6H_2_O (8.8), CaCl_2_·2H_2_O (0.05), AMPSO (*N*-(1,1-dimethyl-2-hydroxyethyl)-3-amino-2-hydroxypropanesulfonic acid) (0.6l), resazurin (0.001), yeast extract (3.0), peptone (3.0), and elemental sulfur (10.0). Trace elements, N–P mixture, and Fe-EDTA solutions were added following the method presented by Holden [80]. For investigation of the proteome during culturing with CO, sodium formate, or soluble starch as substrates, modified-M1 (MMI) media [81] containing (g/L) NaCl (35.0), KCl (0.7), MgSO_4_ (3.9), CaCl_2_·2H_2_O (0.4), NH_4_Cl (0.3), Na_2_HPO_4_ (0.15), NaSiO_3_ (0.03), NaHCO_3_ (0.5), cysteine·HCl (0.5), resazurin (0.001), and either CO (100% in the head space), starch (5.0), or formate (5.0) was employed. After autoclaving, the medium was transferred into an anaerobic chamber (Coy Laboratory Products, Grass Lake, MI, USA) and reduced by adding 0.005% (*v*/*v*) 5% (*w*/*v*) Na_2_S·9H_2_O, 1 ml/l Holden’s trace elements [80], and 1 ml/l Balch’s vitamin solution [82]. The initial pH of the media was adjusted to 8 at room temperature. *T. onnurineus* NA1 was cultured separately in four different batches for biological reliability. Culture procedures were generally the same as reported earlier [14]. Based on previous kinetic analysis of growth and H_2_ production on all four substrate, proteomic analysis was therefore conducted on samples harvested 16 h (sulfur and formate) or 20 h (CO and starch) for cells grown on different substrates.

### 3.2. Protein Preparation and Enzyme Digestion

All procedures were carried out essentially as described previously [30]. Briefly, cells were resuspended in 20 mM Tris-HCl buffer (pH 8.0) and then disrupted using Sonics Vibra Cell (Sonics & Materials INS, Newtown, CT, USA) at 30 amplitudes for 1 min. After centrifugation at 18,000 rpm for 1 h, proteins in the supernatant were separated by 12% SDS-PAGE. The gels were then stained with Coomassie Brilliant Blue R250 (Sigma, St. Louis, MO, USA), and the protein bands were cut out according to molecular weight. Following reduction and alkylation of the cysteines, proteins in the sliced gels were digested in trypsin solution for 12–16 h at 37 °C. Digested peptides were extracted with a solution consisting of 50 mM ammonium bicarbonate, 50% acetonitrile, and 0.1% trifluoroacetic acid, and then lyophilized in a vacuum concentrator. Dried tryptic peptides were stored at −80 °C until LC-MS/MS analysis.

### 3.3. Analysis by Nano-UPLC-MS^E^ Tandem Mass Spectrometry and Quantitative Analysis

The prepared peptide mixtures were desalted using a solid phase Oasis HLB C18 microElution plate (Waters Corporation, Milford, MA, USA), and then used for LC–MS/MS analysis. The separations were performed on a 75 μm × 50 mm nano-ACQUITY UPLC 1.7 μm BEH300 C18 RP column and a 180 μm × 20 mm Symmetry C18 RP 5 μm enrichment column, using a nano-ACQUITY Ultra Performance LC Chromatography System (Waters Corporation). The desalted tryptic peptides (5 μL) were loaded onto the enrichment column with mobile phase A (3% ACN in water with 0.1% formic acid). A step gradient was then used at the flow rate of 300 μL/min. The gradient included a step using 3%–40% mobile phase B (97% ACN in water with 0.1% formic acid) over 95 min, 40%–70% mobile phase B over 20 min, and a sharp increase to 80% B within 10min. Sodium formate (1 μmol/min) was used to calibrate the TOF analyzer in the range of *m*/*z* 50–2000, and [Glu1]-fibrinopeptide (*m*/*z* 785.8426) was used at a flow rate of 600 nL/min for lock mass correction. The continuum LC–MS^E^ data were processed and analyzed using the IDENTITY^E^ algorithm. Proteins were identified by searching the *T. onnurineus* NA1 database on the NCBI website (https://ncbi.nlm.nih.gov) using the following parameters: peptide tolerance, 100 ppm; Fragment tolerance, 0.2 Da; missed cleavage, 1; and variable modifications, carbamidomethylation at cysteine. Peptide identification was performed using the trypsin digestion rule with one missed cleavage. All proteins identified based on the IDENTITY^E^ algorithm were kept within >95% probability [30,31]. The false positive rate for protein identification was set to 5% in the databank search query option, based on the automatically generated reverse database of PLGS version 2.3.3. Protein identification was also based on the assignment of at least two peptides with seven or more fragments.

## 4. Conclusions

To the best of our knowledge, this is the first detailed proteomic investigation of sulfur metabolism in hyperthermophilic archaea. By using a comparative proteomic strategy, an overview of the complicated processes behind adaptation to growth on sulfur was presented. Most of the enzymes putatively involved in the sulfur metabolism of *T*. *onnurineus* NA1 were detected, and quantitative information on their relative abundance in cells grown under different substrate culture conditions was obtained. Quantitative proteome analysis using 1D-SDS PAGE coupled with nano-UPLC-MS^E^ is an important tool to increase the understanding of the complex interplay between hydrogen and sulfur metabolism. This work provides information regarding the molecular regulation of hydrogen *versus* sulfur metabolism in archaeal cells, through analysis of hydrogen producing *versus* non-hydrogen producing (sulfur-rich) cultures. In line with the genetic and functional genomics studies on *Thermococcus* sp. and *P. furiosus*, we hereby provide supportive evidence integrating the results from proteomics and genomics in an endeavor to characterize the metabolic switch between sulfur and H_2_ metabolism.

The protein expression profile of *T*. *onnurineus* NA1 under sulfur culture conditions revealed the up-regulation of sulfur-regulated proteins, as well as of a number of proteins involved in electron transfer that could be essential for supplying reductants to the energy-demanding sulfur (S°) reduction process. In contrast, a large number of proteins required for hydrogen production and oxygen detoxification were strongly down-regulated in sulfur-grown cultures. Our comparative proteomic analysis revealed several new aspects of metabolic process in response to sulfur. Furthermore, there were numerous proteins up- and down-regulated uniquely in response to sulfur stress. These proteins had diverse functions, which suggests a complexity of regulation consistent with the metabolic diversity which distinguishes *T*. *onnurineus* NA1 from other genera in the hyperthermophilic archaea. In particular, growth on sulfur resulted in the up-regulation of several proteins associated with the CO_2_ fixation pathway. Our data suggest that *T*. *onnurineus* NA1 can incorporate CO_2_ into cellular material via the incomplete rTCA cycle when hydrogen sulfide is available as an energy source. The most striking finding was that the expression of several ROS response proteins was greatly reduced in sulfur-grown cells, which likely leads to a decrease in the productivity of H_2_. It is also intriguing to note that enzymes involved in the sulfur assimilation pathway, as well as cysteine desulfurase, were found to be the major down-regulated proteins in comparative proteomic analysis.

The proteomic results obtained herein also provide insight into the strategy of *T.onnurineus* NA1 cells in adaptation to sulfur-available environments, accomplished by regulation of the major metabolic processes through changes in protein abundance. This study will contribute to gaining a better understanding of the mechanisms and physiology of *T*. *onnurineus* NA1 cells in response to sulfur, in addition to opening novel avenues for the identification of suitable targets for genetic manipulation that may lead to the development of more effective and sustainable H_2_ production.

## Supplementary Materials

Supplementary materials can be found at http://www.mdpi.com/1422-0067/16/05/9167/s1.
