# Supplementary material for: Proteomic Insights into Sulfur Metabolism in the Hydrogen-Producing Hyperthermophilic Archaeon Thermococcus onnurineus NA1
Source: Int J Mol Sci. 2015 Apr 23;16(5):9167–95. doi: 10.3390/ijms16059167 (PMC4463584; doi:10.3390/ijms16059167)
Supplement: Supplementary file 1 [file ijms-16-09167-s001.zip › ijms-77670-Supplementary Information-figure.pdf]

Supplementary Information

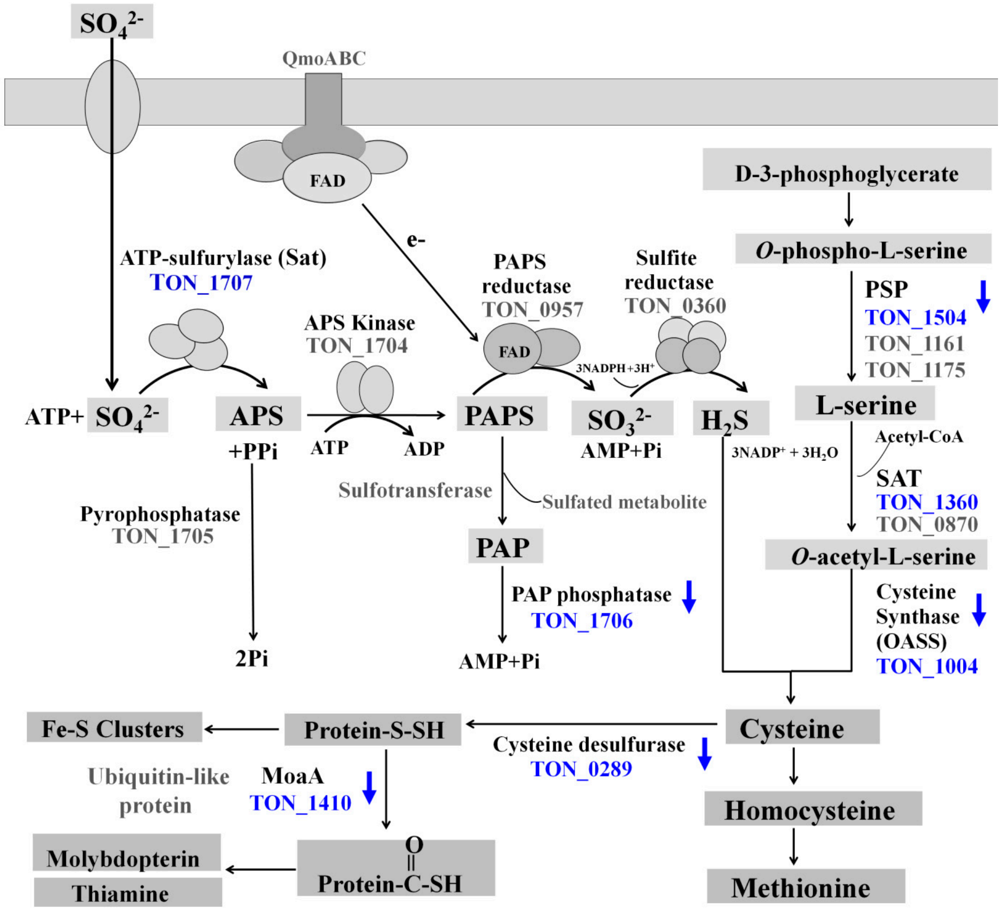

Figure S1. Proposed sulfur assimilation pathway in *Thermococcus onnurineus* NA1. Sulfate is transported into the cell and then reduced to sulfide. Sulfide then reacts with *O*-acetylserine to generate cysteine, which serves as the central sulfur donor for the biosynthesis of other sulfur compounds. For the biosynthesis of other sulfur compounds, cysteine donates sulfur to a conserved Cys residue of cysteine desulfurase to generate a persulfide group, which then functions as the proximal sulfur donor for the biosynthesis of Fe–S clusters. However, the sulfur-carrier protein(s) involved in the Fe–S cluster assembly have yet to be understood. For the biosynthesis of thiamine and molybdopterin, the persulfide group of cysteine desulfurase donotes sulfur to the *C*-terminal Gly of ubiquitin-like proteins such as MoaA to generate a thiocarboxylate group. Enzymes donw-regulated during growth on sulfur are indicated with blue arrows.
